# Supplementary material for: End Sequence Analysis Toolkit (ESAT) expands the extractable information from single-cell RNA-seq data
Source: Genome Res. 2016 Oct;26(10):1397–410. doi: 10.1101/gr.207902.116 (PMC5052061; doi:10.1101/gr.207902.116)
Supplement: Supplemental Material [file supp_gr.207902.116_Supp_Materials.tar.gz › ESAT-esat_v0.1/doc/html/classbroad_1_1pda_1_1enrichment_1_1_enrichment_metric.html]

ESAT: broad.pda.enrichment.EnrichmentMetric Class Reference


ESAT


- Main Page
- Related Pages
- Packages
- Classes
- Files

- Class List
- Class Index
- Class Hierarchy
- Class Members

All Classes Namespaces Files Functions Variables Enumerator Pages

- broad
- pda
- enrichment
- EnrichmentMetric

Public Member Functions |
Protected Attributes |
List of all members

broad.pda.enrichment.EnrichmentMetric Class Reference

|  |  |
| --- | --- |
| Public Member Functions | |
|  | EnrichmentMetric (String name, String dataType1, String dataType2, String statisticName, String method, double statistic, double pvalue, int totalCount, int regionCount) |
|  | |
| Set< String > | getDataTypes () |
|  | |
| String | getDataType1 () |
|  | |
| String | getDataType2 () |
|  | |
| String | getMethod () |
|  | |
| String | getSubmethod () |
|  | |
| double | getStatistic () |
|  | |
| double | getPValue () |
|  | |
| String | toString () |
|  | |
| String | getId () |
|  | |

|  |  |
| --- | --- |
| Protected Attributes | |
| String | name |
|  | |
| String | statisticName |
|  | |
| double | statistic |
|  | |
| int | totalCount |
|  | |

## Detailed Description

All enrichment metrics should inherit this class. Could use the PICARD base for potentially easy output

Author
:   engreitz

## Constructor & Destructor Documentation

|  |  |  |  |
| --- | --- | --- | --- |
| broad.pda.enrichment.EnrichmentMetric.EnrichmentMetric | ( | String | *name*, |
|  |  | String | *dataType1*, |
|  |  | String | *dataType2*, |
|  |  | String | *statisticName*, |
|  |  | String | *method*, |
|  |  | double | *statistic*, |
|  |  | double | *pvalue*, |
|  |  | int | *totalCount*, |
|  |  | int | *regionCount* |
|  | ) |  |  |

## Member Function Documentation

|  |  |  |  |  |
| --- | --- | --- | --- | --- |
| String broad.pda.enrichment.EnrichmentMetric.getDataType1 | ( |  | ) |  |

|  |  |  |  |  |
| --- | --- | --- | --- | --- |
| String broad.pda.enrichment.EnrichmentMetric.getDataType2 | ( |  | ) |  |

|  |  |  |  |  |
| --- | --- | --- | --- | --- |
| Set<String> broad.pda.enrichment.EnrichmentMetric.getDataTypes | ( |  | ) |  |

|  |  |  |  |  |
| --- | --- | --- | --- | --- |
| String broad.pda.enrichment.EnrichmentMetric.getId | ( |  | ) |  |

Here is the caller graph for this function:

|  |  |  |  |  |
| --- | --- | --- | --- | --- |
| String broad.pda.enrichment.EnrichmentMetric.getMethod | ( |  | ) |  |

|  |  |  |  |  |
| --- | --- | --- | --- | --- |
| double broad.pda.enrichment.EnrichmentMetric.getPValue | ( |  | ) |  |

|  |  |  |  |  |
| --- | --- | --- | --- | --- |
| double broad.pda.enrichment.EnrichmentMetric.getStatistic | ( |  | ) |  |

|  |  |  |  |  |
| --- | --- | --- | --- | --- |
| String broad.pda.enrichment.EnrichmentMetric.getSubmethod | ( |  | ) |  |

|  |  |  |  |  |
| --- | --- | --- | --- | --- |
| String broad.pda.enrichment.EnrichmentMetric.toString | ( |  | ) |  |

Here is the call graph for this function:

## Member Data Documentation

String broad.pda.enrichment.EnrichmentMetric.name

protected

double broad.pda.enrichment.EnrichmentMetric.statistic

protected

String broad.pda.enrichment.EnrichmentMetric.statisticName

protected

int broad.pda.enrichment.EnrichmentMetric.totalCount

protected

---

The documentation for this class was generated from the following file:

- java/broad/pda/enrichment/EnrichmentMetric.java


---

Generated on Fri Aug 15 2014 15:10:59 for ESAT by  

 1.8.7
